# Fluoxetine and Suicide Rates: Suicide and the Economy

**DOI:** 10.1371/journal.pmed.0030501

**Published:** 2006-11-28

**Authors:** Carlos A Camargo, Daniel A Bloch

**Affiliations:** Stanford University School of Medicine, Stanford, California, United States of America

**Figure 1:**
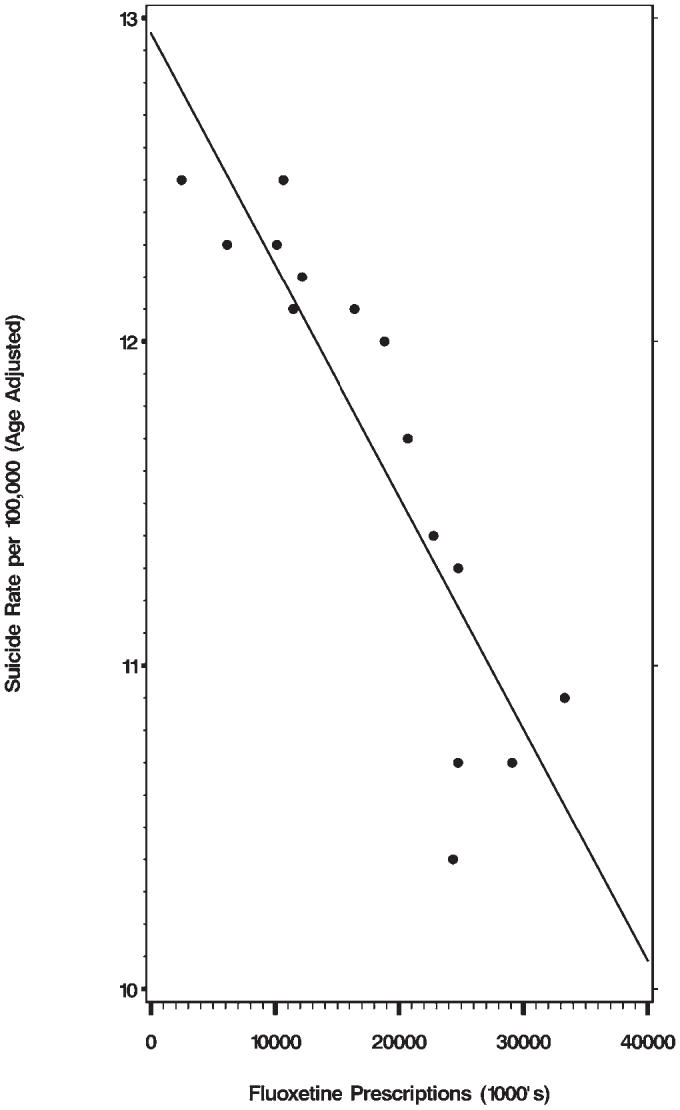
Correlation between Suicide Rates and Fluoxetine Prescriptions

We wish to comment on the paper by Milane et al. [1] and also refer to the Perspective by Baune and Hay [2] in the June issue of *PLoS Medicine* on the effect of fluoxetine prescriptions on the suicide rate in the United States. Milane et al. examined two sets of variables: the number of prescriptions for fluoxetine in the United States, and the Census Bureau mortality tables with the age adjusted suicide rates for the years 1988 to 2002. The date 1988 is chosen because in that year fluoxetine was introduced in the US. The authors report that the Spearman correlation coefficient between the two sets of variables equals −0.92 with a *p*-value of less than 0.001. The less suicides, the more tablets of fluoxetine are prescribed, or vice versa. The least-squares regression line is displayed in Figure 1.

From this simple association they build an elaborate edifice, predicting what the suicide trends would have been had fluoxetine not been prescribed, and they calculate figures for “the thousands of lives saved” for both men and women...even though it is not known how many of these prescriptions were for men or for women, whether the patients took the tablets or not, or for how long they took the medication. In addition, the baseline period used to calculate the suicide trend, and thus to predict the future, was arbitrary: from 1960 to 1987, when the suicide rates had a slight gradual increase in the 1970s. Had they used the period 1950 to 1987, a different “trend” would have been obtained, since the suicide rates decreased during the economic expansion of the 1950s [3].

It is widely known that one cannot infer causality simply based on statistical association. Baune and Hay pointed this out and wrote: “In a study like this, it is also important to consider other potential explanations for the fall of suicide rates, such as improvements in the economy...” In this letter we report on the association of other variables with the suicide rates, for we find that the most glaring defect of the Milane et al. article is the total absence of analysis to address likely confounding by many other factors.

Suicide is the final outcome of many conditions, and there have been, for many decades, scholarly articles indicating the many risk factors which increase the likelihood of suicide: poverty, loss of employment, and several other economic indicators have been shown to have a strong effect upon suicide rates. For example, during the Great Depression of the 1930s the rate of suicide rose significantly, and fell when the economy improved and unemployment decreased in the 1940s. On this matter, the literature is quite clear and the references abundant [4–8]. In the 1990s there was a very substantial and prolonged improvement of the US economy [9], which could partially explain a lowering of the suicide rate. We have chosen three economic indicators for the period from 1988 to 2002 and correlated them with the suicide rate, using the Spearman correlation coefficient to quantify the strength of the association. The yearly data for the suicide rates and numbers of fluoxetine prescriptions, for the three economic indicators (Dow Jones average, food stamp rate, and unemployment rate) and for the property crime and burglary rates are all contained in Table 1. The findings are not surprising: The unemployment rate during those years has a strong positive correlation with the suicide rate: *r* = 0.62, *p* = 0.014.

**Table 1:**
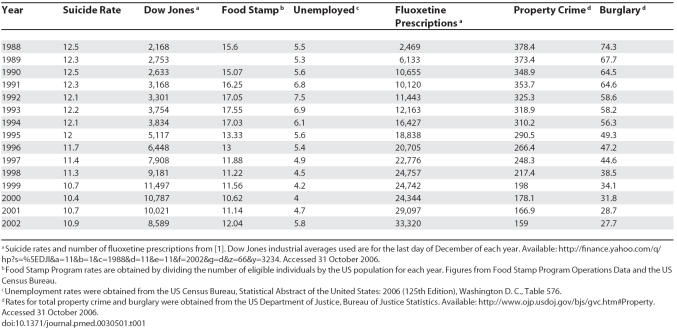
Raw Data by Year

The percentage of the US population eligible for the Food Stamp Program, a reasonable indicator of poverty rates, has a stronger positive correlation with the suicide rates: *r* = 0.84, *p* = 0.0002.

**Figure 2:**
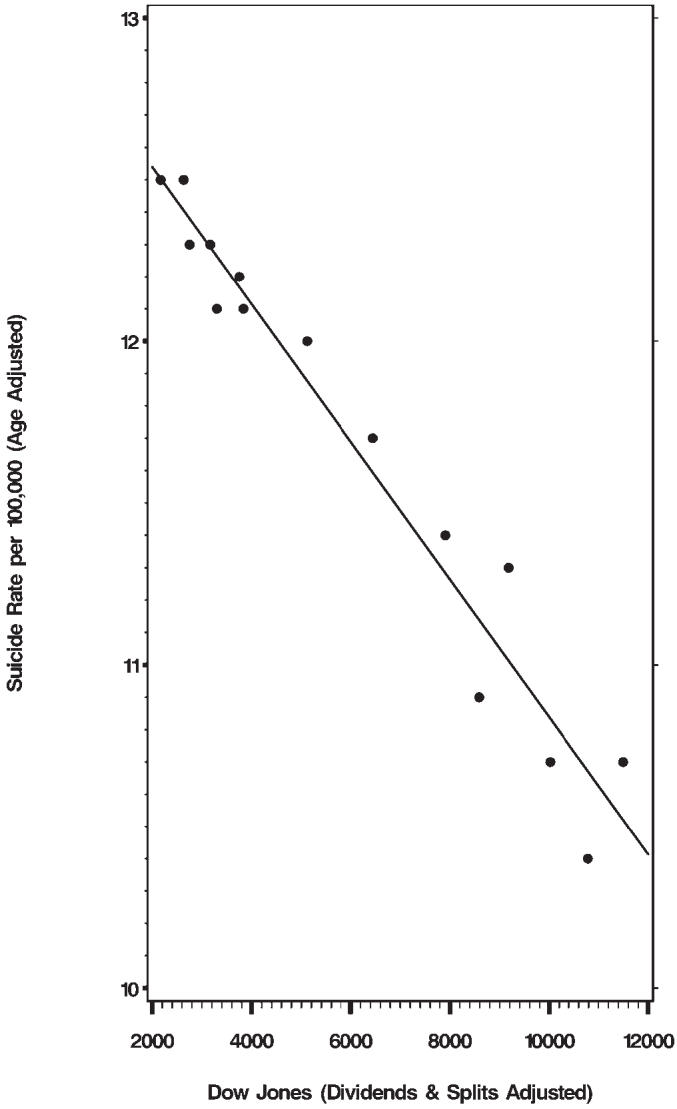
Correlation between Suicide Rates and the Dow Jones Average

The Dow Jones industrial average for each of those years, when compared with the suicide rate of the US population, gives an even stronger (negative) correlation: *r* = −0.98, *p* < 0.0001 (see Figure 2).

**Figure 3:**
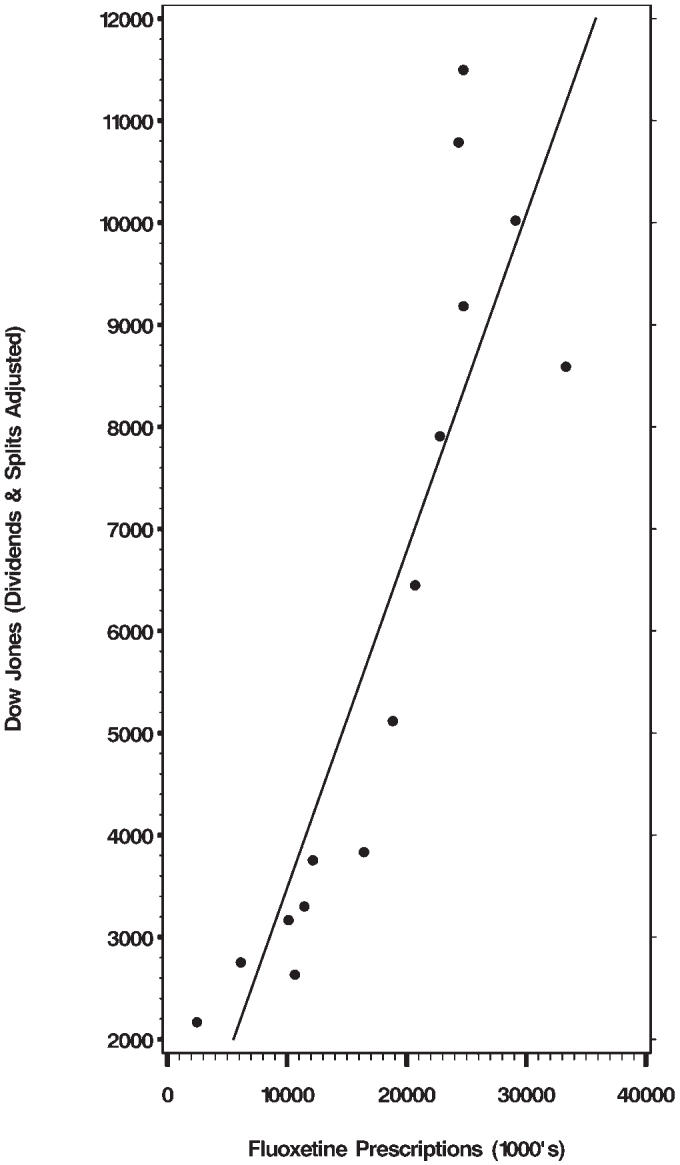
Correlation between Fluoxetine Prescriptions and the Dow Jones Average

We also calculated the correlation between fluoxetine prescriptions and the Dow Jones average. Not surprisingly, there is a very strong positive correlation: *r* = 0.925, *p* < .0001 (see Figure 3).

We doubt that many will advance the thesis that the increasing sales of fluoxetine were, somehow, one of the causes of the rise of the Dow Jones index. In fact, if the number of fluoxetine prescriptions is correlated with any variable that also steadily increased, or decreased, during all those years (1988–2002), a statistically significant association is most likely to be demonstrated. For example, the rate of crimes against property, obtained from the US Department of Justice, for the period 1988–2002 also exhibits a very high negative correlation with the fluoxetine prescriptions: *r* = −0.99, *p* < 0.0001. The rate of burglaries does also: *r* = −0.99, *p* < 0.0001. These relationships are not causal. Most scholars would relate the decrease in crime rates to the improvement of the economy during those years, rather than to increased sales of fluoxetine. The Spearman correlation coefficients for all possible pair-wise comparisons are contained in Table 2.

**Table 2:**
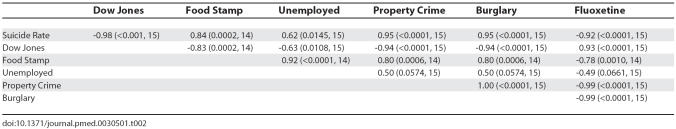
Spearman Correlation Coefficients (*p*-Value, Sample Size)

Given these findings, we decided to explore the relationship of suicide rates with both fluoxetine prescriptions and Dow Jones averages as potentially associative factors in a single multivariate model. Results are displayed in Table 3. This allowed us to assess the association between fluoxetine and suicide adjusting for the Dow Jones, an economic indicator. Statistically, the association is quantified with a “partial” Spearman correlation coefficient. With this analysis the fluoxetine association was not significantly correlated with the suicide rate: fluoxetine had an adjusted Spearman correlation of −0.18 (*p* = 0.54) whereas the adjusted Dow Jones correlation remained high at −0.88 (*p* < 0.0001).

**Table 3:**
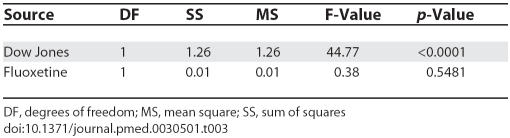
Type III Analysis of Variance Table for Regression of Suicide Rate on Dow Jones Averages and Number of Fluoxetine Prescriptions

In conclusion, we believe that there is little likelihood that the increasing sales of fluoxetine from 1988 to 2002 were the cause of the modest decrease in the suicide rate during those years. It appears more likely that factors such as those connected with the sustained economic recovery of the 1990s were responsible.
